# Seeking the neural representation of statistical properties in print during implicit processing of visual words

**DOI:** 10.1038/s41539-023-00209-3

**Published:** 2023-12-16

**Authors:** Jianyi Liu, Tengwen Fan, Yan Chen, Jingjing Zhao

**Affiliations:** 1 School of Psychology, Shaanxi Normal University, and Key Laboratory for Behavior and Cognitive Neuroscience of Shaanxi Province, Xi’an, China; 2 Key laboratory of Adolescent Cyberpsychology and Behavior (CCNU), Ministry of Education, Wuhan, China; 3 Key laboratory of Human Development and Mental Health of Hubei Province, School of Psychology, Central China Normal University, Wuhan, China

**Keywords:** Language, Reading

## Abstract

Statistical learning (SL) plays a key role in literacy acquisition. Studies have increasingly revealed the influence of distributional statistical properties of words on visual word processing, including the effects of word frequency (lexical level) and mappings between orthography, phonology, and semantics (sub-lexical level). However, there has been scant evidence to directly confirm that the statistical properties contained in print can be directly characterized by neural activities. Using time-resolved representational similarity analysis (RSA), the present study examined neural representations of different types of statistical properties in visual word processing. From the perspective of predictive coding, an equal probability sequence with low built-in prediction precision and three oddball sequences with high built-in prediction precision were designed with consistent and three types of inconsistent (orthographically inconsistent, orthography-to-phonology inconsistent, and orthography-to-semantics inconsistent) Chinese characters as visual stimuli. In the three oddball sequences, consistent characters were set as the standard stimuli (probability of occurrence *p* = 0.75) and three types of inconsistent characters were set as deviant stimuli (*p* = 0.25), respectively. In the equal probability sequence, the same consistent and inconsistent characters were presented randomly with identical occurrence probability (*p* = 0.25). Significant neural representation activities of word frequency were observed in the equal probability sequence. By contrast, neural representations of sub-lexical statistics only emerged in oddball sequences where short-term predictions were shaped. These findings reveal that the statistical properties learned from long-term print environment continues to play a role in current word processing mechanisms and these mechanisms can be modulated by short-term predictions.

## Introduction

Reading is an important social skill that enables us to extract the wisdom of others from a range of symbols. These symbols are visual words with various orthographic features. Proficient reading requires the assimilation of statistical regularities present in the writing system^1^. This statistical structure includes both information at the sub-lexical level, such as orthographic regularities (e.g., frequency and/or legality of consonant doublets in alphabetic language; the frequency of a radical occurs in a given location within characters in Chinese), orthography-to-phonology (O-P) consistency (e.g., consistent body-rime correspondences such as pill, mill, and still; “inconsistent” words such as pint) and orthography-to-semantics (O-S) consistency^2–6^, and information at the lexical level such as word frequency (i.e., the rate of occurrence of an orthographic form) that also captures some of the statistical structure in the mappings from O-P and O-S at the whole-word (lexical) level^1^.

The acquisition of such regularity information is considered to depend on statistical learning^1^ (SL), which refers to learning based on some aspect of the statistical structure of input elements, primarily their frequency, variability, distribution, and co-occurrence probability^7^. SL can occur implicitly and has been observed to be demonstrated across a variety of both linguistic and nonlinguistic contexts^8–13^. The investigation into the role of SL in language acquisition originated from the seminal study conducted by Saffran and her colleagues^14^, which demonstrated that infants possess sensitivity to transitional probabilities (TPs) of syllables within a continuous speech stream. It was seen as providing a viable explanation for identifying word boundaries. In the many hundreds of studies that followed the original auditory TP learning task by Saffran et al.^14^, researchers often tailored the task’s parameters (including the adaptation of nonlinguistic domain and visual modality) to address closely related questions^15^. TP is a type of conditional probability, which essentially reflects the raw frequency of co-occurrence^16,17^. This also makes it limited by the coverage of conditional probability, that is, TP can mainly explain the learning of adjacent regularities (e.g., one syllable predicted the syllable directly following). However, SL applies not only to the acquisition of adjacent regularities, but also to the learning of nonadjacent regularities – regularities that exist over an intervening element (e.g., refs. ^18,19^). Distributional SL account for the learning of non-adjacent relations, which is termed for sensitivity to those aspects of the statistical structure of the input that capture the frequency and variability of exemplars in the input^7,17^. In contrast to conditional SL, which focuses on the acquisition of local statistical structure such as TP learning, distributional SL places greater emphasis on acquiring global statistical structure. These two types of SL may have different contributions to learning different language knowledge, for example, word segmentation depends more on conditional SL, while orthographic and morphological regularities of written words rely more on distributional SL^3^.

Classical SL experiments represented by TP learning (e.g., auditory triplet learning; visual triplet learning) and artificial grammar learning tasks have typically considered learning on the timescale of minutes. However, SL has a continuous learning trajectory that begins with low-level coding of uncertainty (for single stimulus tokens) and ends with long-term accumulated knowledge of the environment^15^. Although there is research evidence to confirm the relationship between these short-term SL effects measured by the classical SL paradigms and reading performance^20–23^. The external validity of this evidence is limited because these studies typically deal with learning of a single type of regularity over a short period of time (see refs. ^7,15^ for detailed discussion). The complexity of the regularities in a given domain, whether a spoken language or a printed text, is often significantly different from these simplified learning problems. A major review recently highlighted the importance of evidence from tasks that tap regularities characteristic of real-world environments across different domains^24^. In fact, there has been a study that has made attempts in this direction^1^. This study used an alternative approach that focused on identifying individual differences in children’s reliance on long-term accumulated statistical regularities as reflected directly in their word naming behavior. The researchers found that the measures of reliance on O-P and O-S had much stronger predictive power than the much weaker correlations observed in correlational studies of “typical” SL tasks and reading outcomes. The authors argued that these results suggest that these more complex regularities are the ones that play a role in reading acquisition, more so than the simplified regularities typically studied in classical SL paradigms. The study of Siegelman et al.^1^ considering the long-term accumulated knowledge of statistical regularities in written language is a new attempt, it can help inform researchers about the subtle regularities that humans are able to assimilate “in the wild”.

So far, there is only circumstantial evidence on whether the distributional statistical properties of print have been implicitly learned by proficient readers through long-term exposure experience to print environments. At the lexical level, the typical evidence is the word frequency effect^25^ (WFE), whereby high-frequency words exhibit processing advantages over low-frequency words across a range of tasks (e.g., word naming, lexical decision, semantic decision; for a review see Brysbaert et al.^26^). In addition, the WFE has also been verified by several ERP studies, the typical example is low-frequency words produce larger N400 amplitudes than high-frequency words (see Kutas & Federmeier^27^, for a review). At the sub-lexical level, a variety of findings make clear that skilled readers read faster and more accurately words with O-P mappings that are more consistent at multiple grain-sizes (e.g., grapheme-phoneme consistency^28,29^; body-rime consistency^30,31^). Additionally, several event-related potential (ERP) studies have validated the consistency effects (low-consistency words evoke larger ERP amplitudes than high-consistency words) of Chinese characters within the time windows of several ERP components^32–35^. However, these results can be explained by different theories (e.g., dual-route model^36^; connectionist model^37^) and therefore cannot be conclusively attributed to the effects of SL. If we can surpass the impact of statistical properties on word recognition and demonstrate that the human brain genuinely decodes these statistical attributes, then we will provide more compelling evidence for long-term SL effects. In order to obtain this critical evidence, we intend to draw momentum from multivariate pattern analysis (MVPA) in cognitive neuroscience. In contrast to traditional univariate analysis techniques (e.g., ANOVA based on ERP amplitudes), MVPA considers the relationship among multiple variables (e.g., channels in EEG), which can capture the information that is not detectable in univariate analysis and improves the sensitivity of identifying differences among experimental conditions^38,39^. The most popular applications of MVPA are decoding (for reviews, see e.g., refs. ^40,41^) and, more recently, representational similarity analysis^42^ (RSA).

RSA is based on the assumption that stimuli (or manipulated features) with more similar neural representations are more difficult to decode, while those with more distinct representations are expected to be easier to decode^38^. By comparing the decodability of all possible pairwise combinations of stimuli, a representational dissimilarity matrix (RDM) is calculated. That is, for each pair of stimuli, the distance between their activation patterns (e.g., the representation vector composed of 64 electrode signals in EEG) is computed using one of several distance metrics (e.g., correlation between the activation patterns or difference in classifier performance^43^). Critically, we can calculate a model RDM based on experimental design in the same way, for example, we can calculate pairwise correlations between all words in a word recognition experiment using the frequency (obtained in the corresponding corpus) of each word as its characteristic. Further, by comparing RDMs from brains and models (e.g., Spearman rank correlation), researchers can know whether brain representations reflect stimulus properties. For data with high temporal resolution such as EEG, a series of RDMs can be created for each time point and used to investigate the temporal dynamics of representations over time.

The Chinese writing system, which has rich quasi-regularity and distributional properties, may provide excellent material for examining the processing of complex statistical regularities in reading^3^. These orthographic distributional regularities are mainly placed among two elements called radicals, one that provides information about how that character is pronounced (phonetic radical), and the other providing information about its meaning (semantic radical). Approximately 80%−90% of Chinese characters are compound characters consisting of these two radicals^44^. For example, in Chinese character 湖/hu:2/ (to lake), the phonetic radical 胡/hu:2/ reveals its pronunciation (/hu:2/), while the semantic radical 氵 indicates its semantic category (water-related concept). In reality, however, many radicals clearly deviate from positional and mapping (i.e., radical-to-phonology and radical-to-semantics mappings) regularities in varying degrees. The obvious feature of the positional distribution of characters is that the majority of them are left-right horizontally structured characters (around 69%; according to ref. ^45^). This, in turn, implies the presence of other structures (positional inconsistent characters). For mapping regularities, approximately 35% of Chinese characters are phonetic inconsistent characters that differ from the common pronunciations of other characters made up of the same phonetic radicals. Furthermore, 12% are semantic inconsistent characters that differ from the common meaning categories of additional characters that are made up of the same semantic radicals^46^. This allows us to better manipulate various statistical attributes of words independently. Specifically, characters that are inconsistent in any one dimension may be consistent in the other dimensions (e.g., 银/yin/, to silver, is inconsistent for common pronunciation, /hen/, but consistent with the meaning category of 钅 as a metal-related concept; 很/hen/, to very, is consistent for common pronunciation, /hen/, but inconsistent with the meaning category of 彳 as a walking-related concept). Therefore, we can simultaneously manipulate the consistency of orthographic, phonological, and semantic within a single Chinese character, and construct the appropriate RDM for each consistency feature.

Assuming that prediction is crucial for SL^47–49^, then predictive coding emerges as an enchanting framework to elucidate the underlying predictive processes. Within the framework of predictive coding, learning is a continuous optimization of a generative model that reflects the world around us and attempts to explain the causes of the sensory inputs^50,51^. This optimization process is achieved by the continuous interaction between top-down flow of predictions and bottom-up flow of prediction errors (the difference between sensory inputs and predictions). Extensive evidence to date indicates that neurophysiological and behavioral responses can unveil musical and linguistic SL effects in the predictive coding framework (e.g., refs. ^52–60^). In neural response, several studies have examined the role of predictions in regulating the intensity of electrophysiological activities. For example, researchers^53–56^ have reported that tones with higher TP (i.e., more-predictable tones) evoked weaker event related potential (ERP) amplitudes compared to tones with lower TP (i.e., less-predictable tones) (see ref. ^61^ for review). In addition, starting from another aspect, several studies^59,60,62–64^ focus on the processing of prediction errors due to SL. These studies explored mismatch negativity (MMN), an ERP differential component interpreted as the neural manifestation of prediction error^65–69^, and reported a “statistical MMN” evoked by probabilistic properties (i.e., TPs) of sounds acquired through SL rather than their acoustical features. The MMN is typically measured with a passive oddball paradigm (employ tasks that are not related to the attributes of the stimuli being explored), in which a series of standard stimuli are interspersed with acoustic deviants (“oddballs”; e.g., sounds differing in pitch, timbre or location^66,70–73^). Within this paradigm, the predictable sounds (standards) are subtracted from the surprising sounds (deviants) to obtain the MMN. As in the statistical MMN experiments, by manipulating the feature types of standard and deviant stimuli, researchers can examine prediction errors for different physical or abstract properties. Substantial evidence has accumulated suggesting that prediction error caused by visual deviants can be reflected in visual MMN (visual counterpart of the auditory MMN). At the outset of vMMN research, studies focused on simple physical property deviances (color, spatial frequency, shape, movement direction, etc^74–77^); later vMMN has been investigated for abstract property deviances (facial emotions, word meaning, phonological categorization, etc^69,78–83^; for a review see ref. ^84^). To date, no vMMN studies have explored the prediction error response related to probabilistic properties of printed words. This raises two important questions. The first is whether distributional statistical regularities in real-world written language are reflected in the neural processing of visual words by skilled readers. If the answer is yes, the second question is whether the neural activities in response to these statistical properties are continuously modulated by prediction error in the current sensory environment.

The present study aims to address these questions and considers the real-world statistical regularities of the Chinese writing system. We designed an experiment using the passive oddball paradigm containing equal probability sequences. For the materials, we carefully selected three consistent Chinese characters and nine inconsistent Chinese characters (see the Materials section). These inconsistent characters were divided into three categories, each with low consistency only in a particular sub-lexical dimension (orthographic, phonological, or semantic). This setting of stimuli is similar to the multi-feature paradigm in auditory MMN studies, where properties of multiple dimensions are manipulated simultaneously in the same stimulus^85,86^. Equal probability sequences were used to provoke the neural activities related to visual words that depended on the participants’ long-term experience. There was no clear predictive evidence in this sequence, and all the characters were presented randomly. Oddball sequences were used to provoke the neural activities related to the same characters when the participants were given clear short-term predictions. In these sequences, consistent characters were repeated with a high probability (*p* = 0.75) and one of the three types of inconsistent characters occasionally appeared with a low probability (*p* = 0.25), giving a total of three oddball sequences. To track the neural representations of various statistical regularities, we performed a time-resolved RSA for the electrophysiological activities of the corresponding characters separately in the oddball (integrating all three oddball sequences) and equal probability conditions. Based on the widely reported robust effects of SL on reading in previous studies, we hypothesize that significant neural representations of statistical information (including word frequency and three types of consistency) can be detected even during implicit visual word processing. Starting from the principle that the volatility of the environment will modulate the intensity of prediction error related neural activities^87,88^, we propose a second hypothesis that sub-lexical statistical information (consistency) will be detected stronger neural representation activities in the oddball condition than in the equal probability condition.

## Results

### Behavioral results

The mean hit rates and mean false alarm rates of the button presses as well as the mean press latencies of correct responses for each experiment are summarized in Table 1. The high hit rates (99%) and low false alarm rates (<1%) indicated that the participants were able to accurately focus their attention on the color change detection task.

**Table 1:** Behavioral results.

Block Type	Mean Hit Rates (Range)	Mean False Alarm Rates	Mean Press Latencies (SD)
Equal probability block	99% (96%–100%)	<1%	369.00 ms (30.58 ms)
IOr oddball block	99% (93%–100%)	<1%	393.26 ms (40.07 ms)
IPh oddball block	99% (93%–100%)	<1%	398.22 ms (37.02 ms)
ISe oddball block	99% (93%–100%)	<1%	396.64 ms (34.82 ms)

*IOr* inconsistent orthographic, *IPh* inconsistent phonological, *ISe* inconsistent semantic.

### RSA based on predictor RDMs

**Fig. 1:**
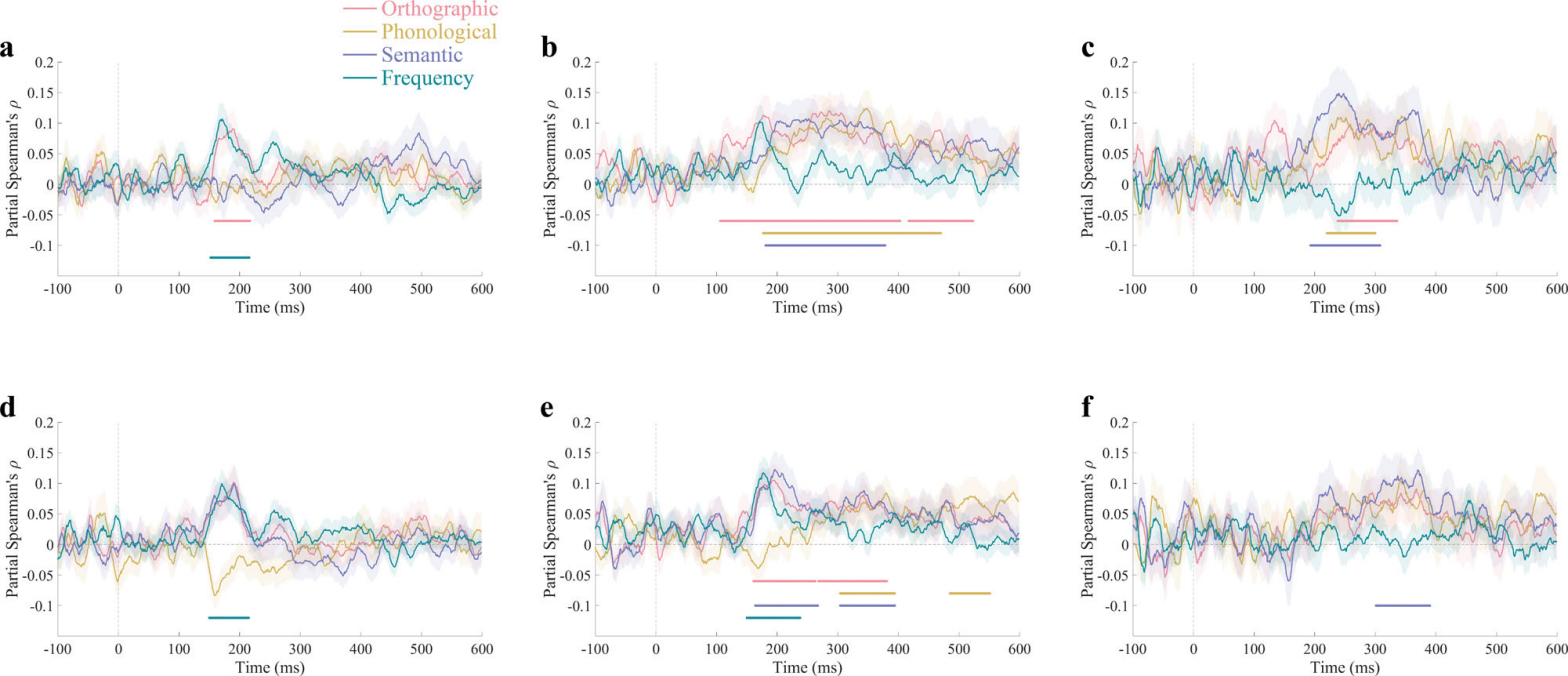
RSA results. Time course of partial Spearman correlations between EEG RDMs and predictor RDMs for orthographic (red), phonological (yellow), semantic (blue), and frequency (green) in equal-probability sequences (**a**), in oddball sequences (**b**), and the difference between them (oddball minus the equal-probability) (**c**). Time course of partial Spearman correlations between EEG RDMs and rating RDMs for orthographic (red), phonological (yellow), semantic (blue), and frequency (green) in equal-probability sequences (**d**), in oddball sequences (**e**), and the difference between them (**f**).

According to the current experimental design, we attempted to detect the classification representations of consistent or inconsistent features in neural activities and obtain corresponding RSA results. Prior to the main analysis, we excluded the interference of phonetic radical categories on neural representation. This is due to the fact that we did not detect significant neural representations of specific phonetic radical in any of the conditions (partial correlation coefficient between neural RDM and radical-control RDM). For characters in the oddball condition, the RSA results revealed the time course of the representations of orthographic consistency (see Fig. 1b, significant time points: 106–403 ms and 417–523 ms), phonological consistency (177–470 ms), and semantic consistency (181–378 ms, all cluster-corrected sign permutation test, cluster definition threshold *p* < 0.05, cluster-corrected significance level *p* < 0.05). The evidence for representations of frequency (lexical statistical information) was absent in the EEG signals of oddball condition. In addition, only frequency (150–215 ms) and orthographic consistency (159–217 ms) representations can be detected in the neural activities corresponding to the characters in equal probability condition (Fig. 1a).

Further information was provided by statistical analysis of the differences in neural representation between the two conditions. The results show that the orthographic (238–336 ms), phonological (220–300 ms) and semantic (193–308 ms) consistency information in oddball condition can be more strongly predicted by neural patterns than those in equal-probability condition (Fig. 1c). It is worth noting that although significant representations of frequency were detected only in equal-probability condition, the intensity of these representations was not statistically different from that obtained in oddball condition. This means that the representation of frequency in oddball condition could be interpreted by stronger representation of sub-lexical statistical information (by partial correlation calculation).

### RSA based on rating RDMs

To examine the generalizability of the results, we performed RSA analysis based on rating RDMs in the same manner. We obtained the representation dynamics of orthographic consistency (161–263 ms and 268–381 ms), phonological consistency (304–394 ms and 485–551 ms), and semantic consistency (164–267 ms and 304–394 ms) in the oddball condition (Fig. 1e). These time regions are basically consistent with the time periods of predictor RDM-based RSA results. We also detected neural representations of frequency in oddball condition from 150 to 238 ms. In the equal probability condition, only frequency is significant (152–216 ms) (Fig. 1d).

The statistical analysis of the representation differences revealed stronger representation of semantic consistency in oddball condition (Fig. 1f). However, although oddball condition also obtained significant representations of orthographic and phonological consistency that were different from equal probability sequence, the differences between these conditions did not reach statistical significance. We believe that this is due to the limited sensitivity of rating RDM detection. The results of frequency on the other hand confirm this view. Because the same frequency RDM was used in the RSA analysis based on predictor RDMs and rating RDMs, the difference between the two is the RDMs controlled in the partial correlation calculation. In the predictor RDM-based analysis, frequency representation is not significant after controlling for orthographic, phonological, and semantic predictor RDM. However, in the rating RDM-based analysis, the representation of frequency was significantly after controlling for orthographic, phonological, and semantic rating RDM. This means that predictor RDMs of various statistical information have stronger detection ability than rating RDM.

### vMMNs results

**Fig. 2:**
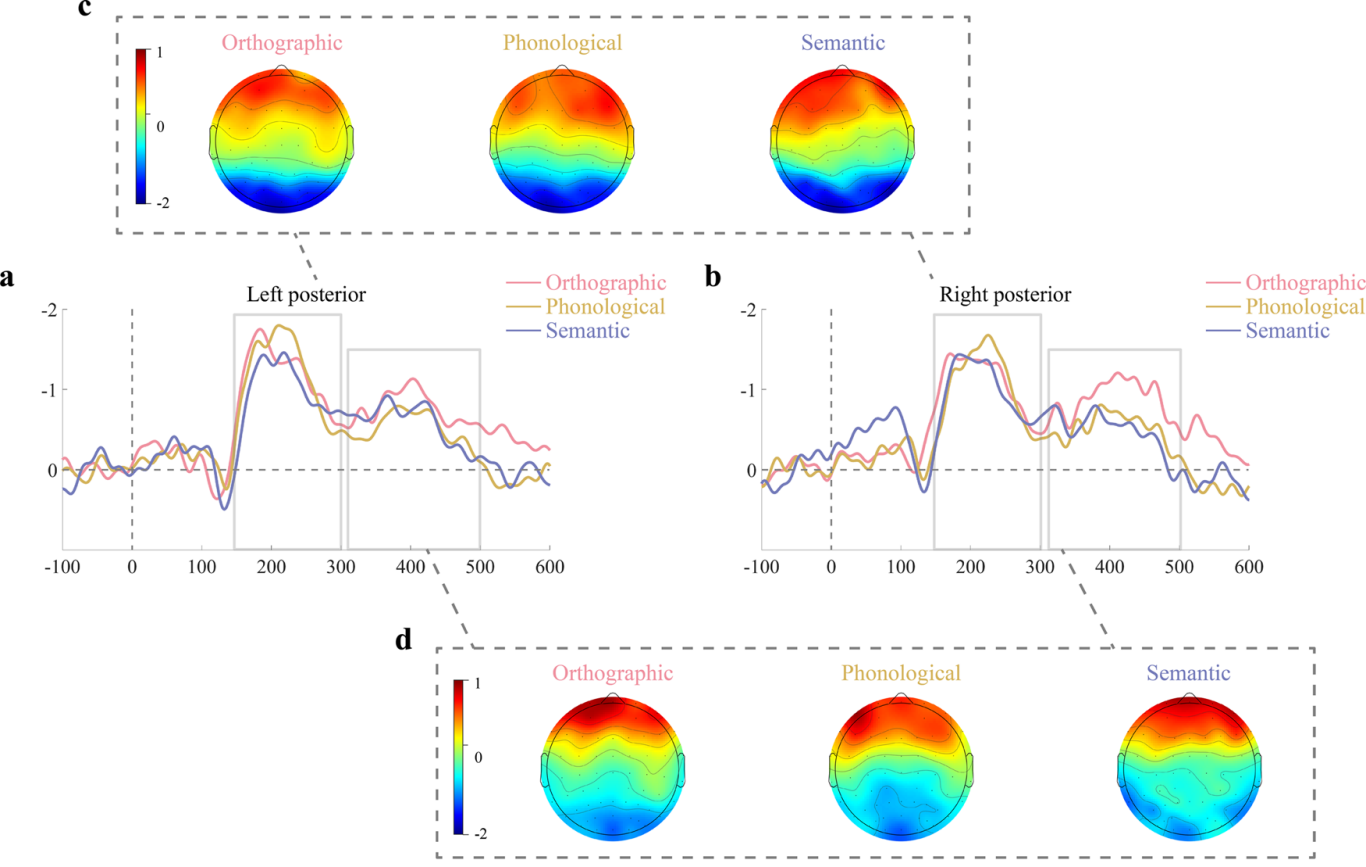
Spatiotemporal distribution of vMMN activities. vMMN waveforms that obtained by subtracting the standard from the deviant characters of each consistency dimension at left (**a**) and right (**b**) ROIs. Scalp topographic maps of vMMNs in two active time periods (150–300 ms (**c**) and 310–500 ms (**d**)).

According to a cluster-based permutation test, the vMMN effect was identified for inconsistent orthographic characters across ROIs in the left and right hemisphere, respectively (Fig. 2a, b). The cluster-based permutation test revealed that there was a significantly stronger negativity for the differential waveform in left (cluster1: sum [t] = –777.98, *p* = 0.0007, effect size = 0.65; cluster2: sum [t] = –643.61, *p* = 0.0013, effect size = 0.85) and right electrodes (cluster1: sum [t] = –776.88, *p* = 0.0003, effect size = 0.76; cluster2: sum [t] = –673.28, *p* = 0.0007, effect size = 0.82) for orthographic-related vMMN. Additionally, a phonological-related vMMN effect was also found in both electrodes that corresponded to a left cluster (cluster1: sum [t] = –678.36, *p* = 0.0007, effect size = 1.03; cluster2: sum [t] = –198.66, *p* = 0.0225, effect size = 0.40) and a right cluster (cluster1: sum [t] = –539.98, *p* = 0.0020, effect size = 0.78; cluster2: sum [t] = –238.30, *p* = 0.0169, effect size = 0.39). Moreover, a vMMN effect was also discovered for inconsistent semantic characters in the left cluster (cluster1: sum [t] = –523.46, *p* = 0.0033, effect size = 0.61; cluster2: sum [t] = –376.73, *p* = 0.0062, effect size = 0.47) and a right cluster (cluster1: sum [t] = –494.44, *p* = 0.0049, effect size = 0.64; cluster2: sum [t] = –361.61, *p* = 0.0095, effect size = 0.57; cluster3: sum [t] = –281.65, *p* = 0.0159, effect size = 0.41). The time range for significant clusters for various vMMNs across different ROI are shown in Table 2. To summarize, these vMMN responses are distributed over two consecutive time windows: 150–300 milliseconds and 310–500 milliseconds. These active time periods of vMMNs are basically consistent with the temporal dynamics of neural representations of sub-lexical statistical information.

**Table 2:** The time window of existing vMMN responses in different ROIs tested by a nonparametric cluster-based permutation test.

vMMN responses	ROIs	Time windows	Cluster mass
inconsistent orthographic	left occipital-temporal	Cluster 1: 313–543 ms Cluster 2: 149–286 ms	–777.98*** –643.61***
	right occipital-temporal	Cluster 1: 310–501 ms Cluster 2: 136–284 ms	–776.88*** –673.28***
inconsistent phonological	left occipital-temporal	Cluster 1: 150–274 ms Cluster 2: 360–439 ms	–678.36*** –198.66*
	right occipital-temporal	Cluster 1: 154–274 ms Cluster 2: 376–474 ms	–539.98** –238.30*
inconsistent semantic	left occipital-temporal	Cluster 1: 159–302 ms Cluster 2: 319–444 ms	–523.46** –376.73**
	right occipital-temporal	Cluster 1: 155–283 ms Cluster 2: 0–111 ms Cluster 3: 359–471 ms	–494.44** –361.61** –281.65*

*p < 0.05, **p < 0.01, ***p < 0.001

## Discussion

The current work explored two important questions closely related to SL: first, whether the statistical properties of real-world print environments as discerned through long-term experience are reflected in the neural processing of words by skilled readers, and second, whether the neural activities in response to these statistical properties are modulated by short-term implicit predictions. Equal probability sequences and oddball sequences were used in this study to detect the neural processing of Chinese characters based on long-term experience and short-term prediction, respectively. We applied RSA to elucidate the neurodynamic pattern of the multidimensional statistical information processing of Chinese characters. First, we found that word (character) frequency (i.e., lexical level statistics) could be rapidly recovered from EEG response patterns in the equal probability condition. This result answers the first question and confirms the existence of implicit neural representations of statistical properties, derived from the long-term textual environment, in word reading. Second, three types of statistical properties at the sub-lexical level (i.e., orthographic, phonological, and semantic consistency) could be extracted in the oddball condition. In addition, the representation strength of these sub-lexical statistics was significantly stronger in the oddball than in the equal probability condition. These differences in the strength of neural representations are consistent with the significant vMMN activities we obtained. These results answer the second question, revealing the existence of prediction error signals driven by prediction related to sub-lexical statistical properties, and confirming the modulation role of prediction precision in the neural representation of these properties.

The present study showed that stable neural representations of word (character) frequency could be detected in the equal probability condition in which there were no clear short-term predictions, and these representations were active for periods of about 150–220 ms after the character was presented. The frequency information implied by the characters could only be interpreted by the statistical distribution characteristics of the long-term reading environment. This provides direct evidence for the effect of SL on visual word recognition and reveals that the statistical properties obtained through long-term SL continues to play a role in current word processing mechanisms. Prior studies^25^ have indicated that the WFE may be part of the indirect evidence for the relationship between SL and reading. Behaviorally, the WFE is a highly replicable and reliable effect that relates to the observation that high-frequency words are processed more efficiently than low-frequency words (e.g., refs. ^89–91^). In the temporal dynamics of neural processing, most studies^92–94^ have found ERP differences between high-frequency (HF) words and low-frequency (LF) words at around 200 ms after word onset. The WFE has also been reported in several recent ERP studies^95,96^ focusing on Chinese characters. Wang and Maurer’s study^95^ found ERP differences at the time interval of 172–253 ms. Another study^96^ used an implicit color decision task to report divergence between the ERPs evoked in response to HF characters and the ERPs evoked in response to LF characters over a time period of 210–222 ms. The ERP time window corresponding to the WFE is consistent with our RSA results. By breaking through the limitation of univariate analysis (the traditional form of ERP analysis), we provide more powerful evidence for the implicit and rapid processing of word frequency information. However, almost no significant neural representation activities in response to sub-lexical level statistical properties were detected in the equal probability condition. This suggests that skilled adult readers may be sensitive to larger-grain size statistical properties (i.e., at the lexical rather than sub-lexical level) in their daily word processing. In fact, this finding is consistent with prior studies of alphabetic languages. Some studies have reported that adults are particularly impacted by body rime rather than grapheme-phoneme level regularities (e.g., refs. ^30,31^). It has been reported that, as humans develop, they become increasingly reliant on O-P regularities at larger grain sizes^29^ (i.e., from the sub-lexical to the lexical level).

Neural representations of various sub-lexical level statistics (i.e., orthographic, phonological, and semantic consistency) become detectable when subjects are exposed to visual inputs that contain clear short-term predictions (i.e., in oddball sequences). In addition, the intensity of these neural representations is significantly stronger than those evoked in the equal probability condition. These findings reveal a short-term plasticity mechanism for neural representations of sub-lexical level statistical properties. We suggest that this plasticity mechanism, as observed in the present experiment, can be appropriately explained by the predictive coding framework^50,65^. In simple terms, predictive coding is an implicit process that creates an internal model of sensory inputs with the aim of minimizing surprise^97^ (i.e., a quantitative formulation of prediction error, which is the negative log probability of a sensory event). In order to minimize the cost (free-energy^50^) of reducing prediction errors, the system assigns different weights to real-time prediction errors according to the variability of the environment (i.e., prediction precision), which causes the same external input to evoke different levels of response depending on the environment in which it is located. This precision setting mechanism can be conceptually understood as a form of meta-learning: learning what is learnable or estimating the predictability of new contingencies^98^. Returning to the current experiment, a lack of effective prediction (i.e., low precision) was observed within the equal probability sequences. All characters were decoded using the established neural processing patterns, corresponding to neural activities in skilled adult readers that are sensitive to lexical rather than sub-lexical statistical properties. However, the situation changed in the oddball sequences, where the presence of unambiguous prediction (i.e., high precision, by repeated exposure to consistent characters) caused any inconsistent information to be evaluated as worthy of learning, thereby activating additional neural activity that was different from the existing processing patterns. This was ultimately reflected in stronger and more explicit neural representations of the various types of sub-lexical statistical properties. In fact, a recent study using the oddball paradigm reported similar findings^88^. That study investigated the neural mechanisms that underpin SL and volatility attuning, and showed that, in stable conditions, SL (as behaviorally assessed) was improved compared to the volatile conditions, prediction errors increased, and there was a greater modulation of neuronal gain, forward connections, and backward connections.

We obtained significant “genuine” vMMN responses within the active time window of neural representations of various forms of sub-lexical statistical properties. The vMMN responses obtained in the oddball paradigm were interpreted as a neural manifestation of the prediction error signal^65,87,99,100^. The prediction error in the current experiment could be clearly attributed to the violation of the consistency category. There are three reasons for this attribution. (1) The vMMNs were calculated via subtraction of the ERPs evoked in response to the equiprobable stimuli from the ERPs evoked in response to the deviant stimuli. The equiprobable and the deviant stimuli were comprised of the same Chinese characters, so that the vMMN cannot be described as involving physical, orthographic, phonological, or semantic differences between them. (2) As the probability of presenting the equiprobable and the deviant stimuli were exactly equal (i.e., 1/4), then the vMMN cannot be explained as a difference in refractoriness between the ERPs evoked by them. (3) The vMMN cannot be explained as a violation of a phonological category or a semantic category since there was no reducible phonetic or semantic category within the deviant or equiprobable characters in the oddball sequence. In summary, we believe that our study defines a class of prediction error responses driven by consistency category violation. These categories of consistency at the sub-lexical level were only available from statistical mappings of a long-term reading environment, so these findings further illustrate the dynamic interaction between short-term plasticity driven by prediction error and long-term experience. We believe that these findings advance the understanding of the mechanisms of SL and provide an interesting perspective on SL from the predictive coding framework.

Finally, let us consider an additional question about categorization. Categorization is a prerequisite for generating vMMN responses, only when the target characteristics of the deviant and standard stimuli are classified as different types, can the deviants produce violations (i.e., surprises) of the predictions that are established by the standard. Categorization is a basic process that includes visual perception, and this process goes beyond the physical characteristics of the stimuli^84^. For example, there are hundreds of colors that fall under the category “blue” even though they have different combinations and values of hue, saturation, and brightness. In the field of character processing, the vMMN effects of word meaning^101^ and phonological^83^ categorization have been investigated. However, the consistency features of concern in the current study are rather special. Phonological consistency, for example, represents how frequently a phonetic radical represents a given sound by calculating the relative proportion of characters with the same pronunciation among those that share the radical (e.g., ref. ^102^). This means that phonological consistency is essentially a continuous variable ranging from 0 to 1, and the other two types of consistency are essentially the same. However, the consistency effect reported in previous studies reflects a dichotomy. These studies often anchor certain most common (i.e., high probability) body rime correspondences as consistent words and others as inconsistent words, and found that consistent words are read aloud faster and more accurately than inconsistent words (e.g., refs. ^31,103^). This consistency effect has also been verified with Chinese characters^102,104,105^. With regard to neural mechanisms, previous fMRI studies^106^ have reported greater activation in the left inferior frontal gyrus, the left temporoparietal (i.e., inferior parietal gyrus and supramarginal gyrus) region, and the left temporal–occipital junction when naming inconsistent characters compared to consistent ones. Additionally, several ERP studies have validated the consistency effects of characters within the time windows of the N170, P200, and N400 components (phonological consistency^32–34^; semantic consistency^35^). In fact, the current experimental design perpetuates the above idea of exploring the consistency effect. Although we did not focus on specific ERP components, our results provide new evidence for the rationality of this line of research. Furthermore, from the perspective of predictive coding, the vMMN activities evoked by consistency category violation may reveal the true state of the brain’s SL product (i.e., neural activities in response to statistical properties contained in the reading environment). In other words, the rich distributional statistical information obtained through SL can be aggregated and reclassified into consistent and inconsistent features and reflected in different neural representation patterns.

We should also note some limitations in the scope and methodology of the current study. First of all, in terms of the scope of current research, although our study was designed to examine the neural representation of statistical properties contained in print learned through long-term exposure. However, the relevant problems are not included in the scope of traditional SL research, and our study is only an exploratory investigation closely related to SL. In addition, previous MMN studies exploring TP learning usually controlled the absolute pitch of the subjects, but the current study did not consider the characteristics of the subjects’ hearing. As no studies have so far explored the relationship between orthographic regularity learning and these hearing characteristics, there are therefore associated potential limitations to the generalizability of our current findings. Finally, in the experimental design, we draw on previous oddball studies that focus on social category information, so that different oddball sequences have the same short-term predictions (by setting the same consistent characters as the standard stimuli). The underlying assumption of this design is that there are no long-term prediction differences between different types of stimuli (i.e., when there is no difference in the probability of presentation, people do not expect to see more of a particular type of stimulus). However, as with most category-based oddball studies, there is not much direct proof of this hypothesis, so we consider it as another potential limiting factor in the explanatory power of the current results.

In summary, our multivariate RSA study demonstrated the contribution of long-term SL to the neural activity related to current word processing. Importantly, we found that the short-term prediction provided by the visual input environment evoked neural representations of sub-lexical statistical properties. In addition, we also obtained significant vMMNs, which indexed an implicit processing mechanism that operates within the predictive coding framework. These findings reveal the link between predictive coding and SL, and confirm the short-term plasticity of neural activities corresponding to long-term SL.

## Methods

### Ethics statement

All participants gave oral and written, informed consent in accordance with procedures that were approved by the ethics committee at the School of Psychology, Shaanxi Normal University (Approval No. HR 2021-05-002). The protocols adhered to the Declaration of Helsinki.

### Participants

Forty-eight healthy young adults were recruited, with three being excluded for excessive EEG artifacts. The final sample of 45 right-handed (via self-report) young adults (mean age = 18.04, SD = 0.80; 35 females; the age range is from 17 to 20) had an average of 12.3 years of education, and all had normal or corrected-to-normal vision. The final sample size surpassed that of similar work^83,101^ using EEG to investigate implicit character recognition during the oddball paradigm and is comparable to other EEG studies using RSA analysis to explore the representation dynamics of language processing^107,108^. Participants were recruited from the undergraduate and postgraduate student population at Shaanxi Normal University and were paid 60 RMB for their participation. All participants reported no speech or hearing problems and had no prior history of neurological or psychiatric abnormalities.

An additional group of 33 paid healthy college students (19 female, mean age = 21.48 years, SD = 2.33 years) were recruited to rate the orthographic consistency, phonological consistency, and semantic consistency (transparency) of each Chinese character we selected. Take the scoring of semantic consistency, one question was asked to measure semantic transparency: “To what extent do you think the radical “X” can represent the meaning of the Chinese character “Y”?”. For example, for the character “洋”, the question was “To what extent do you think the radical “氵“ can represent the meaning of the Chinese character “洋“ ?”. In a similar way, each dimension of consistency was measured on a seven-point scale, with 1 = totally inconsistent and 7 = totally consistent (examples of other questions are in the supplementary notes).

### Materials

**Fig. 3:**
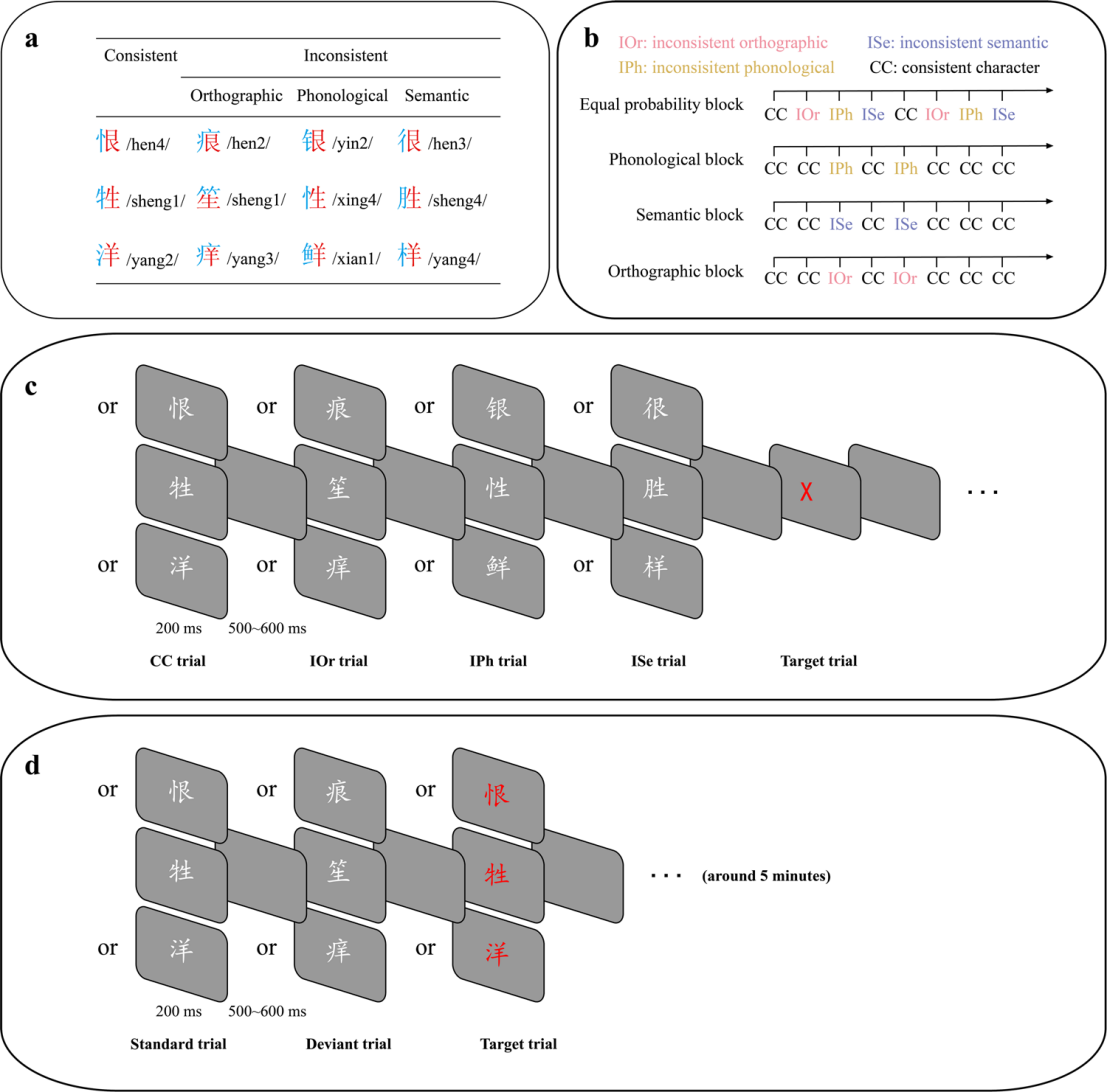
Illustration of the experimental procedure. **a** Details of the selected Chinese characters. **b** Examples of presentation settings for consistent and inconsistent characters in different blocks. **c** Schematic depiction of the color-change judgment task in the equal probability block. **d** Schematic depiction of the color-change judgment task in the oddball block. Abbreviations: CC consistent characters, IOr inconsistent orthographic characters, IPh inconsistent phonological characters, ISe inconsistent semantic characters.

There were three different sets of Chinese phonograms selected. In Chinese, a character is generally made up of a semantic radical and a phonetic radical, known as “phonograms”. For example, the character “牲” consists of a semantic radical “牜” and a phonetic radical “生”. Each phonogram set in the present study has a fixed phonetic radical and four different semantic radicals, it enables us to quantify orthographic consistency features based on phonetic radical (Fig. 3a). In the second row in Fig. 3a, for example, the phonetic radical in all characters is “生”, while the semantic radicals are “牜”, “”, “月” and “忄”, respectively. Thus, each stimulus set contained four characters (each row in Fig. 3a), for a total of 12 characters.

Semantic radicals are generally on the left side of characters, and phonetic radicals are on the right, which is the main orthographic rule in the majority (63%) of phonograms (45). Semantic radicals usually provide semantic information of characters, while phonetic radicals provide phonological clues. For example, in the Chinese character “牲”, the semantic radical “牜” is on the left side, while the phonetic radical “生” is on the right. The meaning of character “牲” is “domestic animals”. This means that it can easily be speculated from the semantic radical “牜”, which refers to “cattle”. Phonologically, the pronunciation of “牲” is “sheng”, which is also highly consistent with the sound of the phonetic radical “生” (sheng). Characters like “牲” are consistent characters. However, there are some characters (i.e., inconsistent characters) that do not follow the orthographic (positional), phonological and/or semantic rules (e.g., “笙”, “性”, “胜”). Accordingly, these 12 Chinese characters were then divided into three consistent and nine inconsistent characters. The nine inconsistent characters were further divided into three categories, including inconsistent orthographic (IOr) characters, inconsistent phonological (IPh) characters and inconsistent semantic (ISe) characters. The three characters in each category come from different stimulus sets. In other words, all four categories (i.e., 1 consistent and 3 inconsistent categories) have the same number of characters and the same phonetic radicals (see Fig. 3a). The inconsistent categories differ from the consistent category with regards to orthographic, phonology and semantics, respectively.

Specifically, compared to the consistent category, IOr characters differ with regards to the structure of characters. That is, phonetic radicals appear on the right side of the character in the consistent category. On the contrary, phonetic radicals in the IOr characters are in the less common position in a character. For example, phonetic radial “生” posits at the right side of a “牲”, but it is at the bottom of the IOr character “笙”. Among all characters that are “艮”, “生” and “羊”, they act as phonetic radicals. The probabilities that “艮”, “生” and “羊” appear on the right side of characters are 72.22%, 50.00% and 72.73%, respectively. On the other hand, the percentages of the character positions for “痕”, “笙” and “痒” are 5.56%, 25%, and 18.18%, respectively (Supplementary Table 1). Furthermore, the position of phonetic radicals in IPh and ISe categories are on the right side, the same as consistent characters.

Similarly, IPh characters differ from consistent characters (CC) with regards to phonological consistency. The pronunciations of CC are high in phonological consistency of corresponding phonetic radicals, while the phonological consistency is low in IPh characters. The phonological consistency is defined as the proportion of a specific pronunciation among all characters that adopt the same phonetic radicals^109^. High consistency refers to the pronunciation of a regular character that is the main pronunciation of all Chinese characters utilizing the specific phonetic radical. In contrast, the pronunciation of IPh characters is a rare sound that corresponds to phonetic radicals. For instance, the pronunciation of “生” and the regular character “牲” are /sheng/, which is the same as most characters that contain “生” (e.g., “笙”, “胜”). However, pronunciation of the IPh character “性” is /xing/, not “/sheng/”. The phonological consistency is 0.39-0.92 for all three CC and is 0–0.28 for the three IPh characters (see Supplementary Table 1 for details). In addition, ISe and IOr characters have the same pronunciation as regular characters (Supplementary Table 1).

Finally, the ISe characters differ from the consistent ones with regards to the transparency of the semantic radical. The CC is high in the transparency of semantic radicals, while the transparency in ISe characters is low. The transparency is defined as the connection between the meaning of the semantic radical, and the meaning of the corresponding character^46^. That is, semantic radicals of CC can reflect the meaning of corresponding characters. However, the meanings of the ISe characters cannot be speculated from the semantic radicals. For example, the semantic radical “牜” (cattle) is related to the meaning of “牲” (livestock). In contrast, the meaning of ISe character “胜” (victory) is much different from that of the corresponding semantic radical “月” (moon). The same as CC, characters in the IPh and IOr categories are high in the transparency of the semantic radical.

### Procedure

The experimental procedure consisted of three oddball blocks and an equal probability block. One of the three categories of inconsistent characters (each category contains three specific characters), in turn, served as the deviant stimuli (dev; probability of occurrence *p* = 0.25; the number of presentations is divided equally among the three specific characters) across different oddball blocks (each block contains 420 trials), while the consistent characters (containing three specific characters) served as standard stimuli (std; *p* = 0.75) (Fig. 3d). In the equal probability block (contains 480 trials), the probability of inconsistent (three categories; named equiprobable stimuli) and consistent characters were the same (*p* = 0.25) (Fig. 3c). Within each block, the trial order was fully randomized, and the order of oddball blocks was also randomized while the equal probability block was implemented at the beginning. For each individual trial, the stimulus was presented for 200 ms, and then a gray image was inserted, lasting for 500–600 ms, at a random time between trials (Fig. 3b). Moreover, the color of the characters may change from white to red at random during some trials (target; *p* = 0.1; may appear on the standard stimuli of the oddball blocks as well as all stimuli of the equal probability block). The task throughout the experiment was to ignore attributes of the character and to press a button with the right thumb as quickly and accurately as possible when red characters (target stimuli) were presented (similar tasks have been widely used in previous studies examining implicit character processing, e.g., refs. ^110,111^). The deviant stimuli in the oddball blocks would not appear twice in a row, and the target only appeared after one standard trial. Participants sat comfortably in an armchair at a distance of 60 cm from the screen, and were given a break for each block that they completed. Using the E-prime software, the images of words were presented within the central visual field (visual angle: horizontally = 2.5°; vertically = 3.8°).

### Behavioral analysis

A participant’s response was counted as a hit if the button was pressed for less than 700 ms after the character color changed. Otherwise, the response was counted as a false alarm. Hit and false alarms (FAs) rates during the color change detection task were analyzed in order to evaluate the degree of commitment to unrelated tasks of the participants.

### EEG recording and preprocessing

Electroencephalography (EEG) signals were recorded through the use of a 64-channel amplifier (ANT Neuro EEGO, Inc.) that was mounted on an electrode cap according to the international 10–10 system. The online reference electrode during the data collection was CPz. The EEG data was digitized at a sampling rate of 1000 Hz, and impedances were kept below 10 kΩ during the experiment.

Offline preprocessing, artifact removal, and data quality assessment was carried out via the Harvard Automated Processing Pipeline for EEG (HAPPE) in MATLAB^112^. A spatially distributed subset of channels providing whole-head coverage was processed (excluding the EOG, M1 and M2 channels). HAPPE’s artifact removal steps included bad channel rejection, removal of 50 Hz electrical noise through CleanLine’s multi-taper approach^113^, and participant artifact rejection (e.g., eye blinks, movement) through wavelet-enhanced ICA with automated component rejection via EEGLAB and the Multiple Artifact Rejection Algorithm^114^. The average (SD) number of independent components (ICs) containing artifacts was 9.3 (3.7). Post-artifact rejection, any channels removed during the bad channel rejection were repopulated through spherical interpolation to reduce spatial bias in re-referencing. After filtered with a 0.1–40 Hz digital Butterworth bandpass filter with a 12 dB/oct roll-off, the EEG data were then re-referenced to the average reference and mean signal detrended. Epochs were created from − 300 ms pre-stimulus to 700 ms post-stimulus for each trial and baseline corrected using the first 100 ms. Any epochs with retained artifact were rejected using amplitude criteria (±100 μV), as in prior research^110^. After epoch rejection, the average (SD) number of trials retained on inconsistent character trials were 31.56 (5.32), 31.72 (5.94), 30.61 (7.26), 29.72 (6.85), 29.33 (6.34), and 30.22 (5.94) for equiprobable IOr, equiprobable IPh, equiprobable ISe, deviant IOr, deviant IPh, and deviant ISe, respectively.

### Representational similarity analysis

To track the representations of individual characters across time, we used RSA^115^. First, we created neural representational dissimilarity matrices (RDMs) for each time point in the EEG epochs (10 ms resolution), reflecting the pairwise dissimilarity of the characters’ brain representations. Second, we modeled the organization of the neural RDMs using Spearman rank correlation coefficients^39,116^, which allowed us to track when representations are explained by the characters’ lexical (frequency) or sub-lexical (containing three dimensions of orthographic, phonological and semantic) statistical information.

#### Neural RDMs

**Fig. 4:**
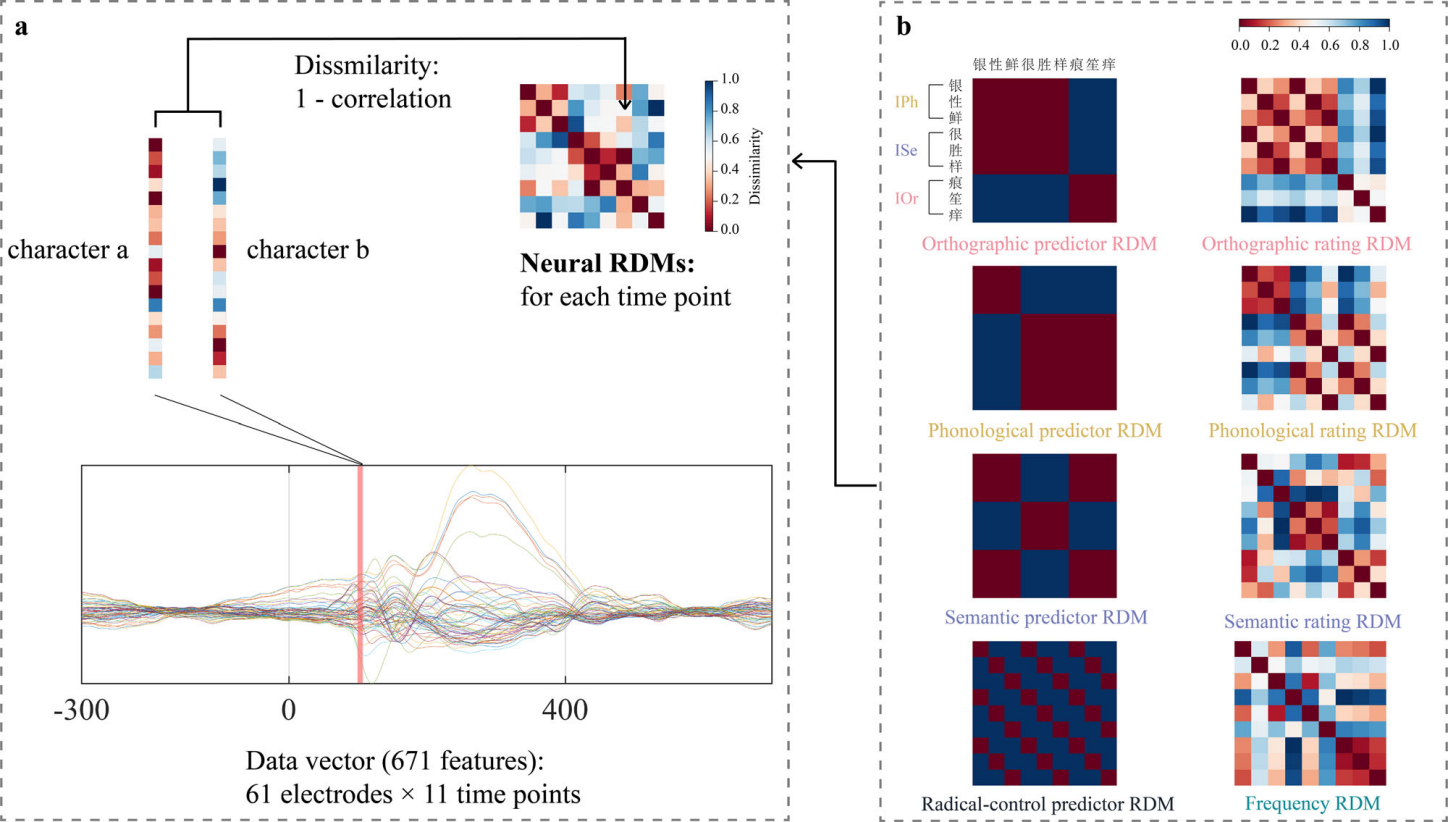
Schematic for representational similarity analyses of EEG data. **a** Neural RDMs are constructed for each data point by comparing pairwise character-specific activations. RDMs are symmetric with a diagonal of zeros, and their size corresponds to the number of inconsistent characters, here 9 × 9. **b** Model RDMs for different dimensional statistical information. Finally, the partial correlation coefficients between neural RDMs and model RDMs was calculated for each subject at each time point to quantify the neural representation strength.

At each time point from 100 ms before stimulus onset to 600 ms after stimulus onset, we correlated the EEG activity between trial pairs (for the nine different inconsistent characters), separately for the oddball condition (put the data of three oddball sequences together) and equal probability condition. This results in a distance value (1- Pearson correlation) that indicates the dissimilarity between character pairs according to brain activity. By repeating this procedure for each pair of characters we constructed a 9 × 9 neural RDM (Fig. 4a). Individual trials were used as input to the RDM calculation. To calculate the time-point by time-point neural RDMs, the vector for the 61 scalp electrodes was concatenated with those of the five preceding and the five succeeding time points, as implemented in CoSMoMVPA^117^. This resulted in a vector length of 671 features reflecting brain activity spanning 10 ms.

#### Model RDMs

We designed two series of model RDMs to explore and validate the representation of different statistical information in the EEG data (Fig. 4b). The first series is a number of RDMs constructed based on the current experimental design. These RDMs will be referred to as predictor RDMs in subsequent texts, and these predictor RDMs include orthographic RDM, phonological RDM, semantic RDM and radical-control RDM (based on the phonetic radical category of the material itself). The above predictor RDMs are 9 × 9 binary RDMs, in which 1 corresponded to a comparison between category character (e.g., consistent vs. inconsistent for the orthographic consistency features), and 0 corresponded to a comparison within category stimuli (e.g., consistent vs. consistent). In addition, we also constructed the frequency RDM according to the word (character) frequency (based on the data from the LCSMCS^118^).

To supplement and validate the results obtained from the predictor RDMs, we constructed another series of RDMs according to the ratings before the formal experiment (from another group of subjects), which resulted in three models of 9 × 9 rating RDMs that corresponded to the orthographic consistency, phonological consistency, and semantic consistency dimensions of our stimuli. Specifically, we calculated the pairwise Euclidean distance between the rating score of each character in each dimension.

#### Representational similarity analysis

The lower off-diagonal of each matrix was extracted as vectors to calculate the Spearman rank correlations between each model and the EEG data. Since some models were correlated, excluding the other models allowed us to separate the contribution of these models from each other^119,120^. In order to explicitly compare lexical and sub-lexical level statistical information models, lexical (frequency) RDM would be excluded when computing a partial correlation between neural RDM and each sub-lexical (orthographic, phonological and semantic) RDMs, and vice versa. We calculated the partial correlation coefficients at each time point for each subject. These partial correlation coefficients served as an indicator of the time course of different statistical information dimensions in the EEG data.

In addition, in order to detect the difference in the representation strength of different statistical information between the oddball condition and the equal probability condition, we calculated the difference of all partial correlation coefficients of each subject under two conditions (oddball minus equal probability).

#### Statistical inference

We performed a non-parametric statistical approach for all RSA results which did not depend on assumptions of the data distributions^121^. Using the maximum cluster size method, significant temporal clusters were defined as adjacent time points that all exceed a statistical cutoff (cluster-inducing threshold). This cutoff was determined through a sign permutation test according to the distribution of t-values from 10,000 permutations of the measured correlation values. The 95th percentile of the t-value distribution was used as the clustering induction threshold of each time point (equivalent to *p* < 0.05, one-sided). To identify significant clusters, we determined the 95th percentile of maximum cluster sizes across all permutations (equivalent to *p* < 0.05, one-sided). This approach provided us with significant temporal clusters in which correlation showed significant effects.

### Visual mismatch negativity (vMMN) analysis

To verify the existence of prediction error responses, we examined vMMN activities using a data-driven approach. The differential waveforms of characters with different inconsistent categories were obtained by subtracting the ERPs of the corresponding deviant stimuli from the ERPs of the corresponding equiprobable stimuli. This method allows the comparison of ERPs that are evoked by the deviant of the oddball sequence to the ERPs that are evoked by physically identical stimuli from a sequence without any particular frequent (standard) stimulus^99^.

The method of equal probability control was suggested in order to deal with repetition effects due to refractoriness that was assumed to be present in the deviant, minus standard activity that was obtained in classical oddball paradigms^122,123^. Activity considered as “genuine” vMMN (i.e., vMMN without stimulus-specific refractoriness effects superimposed) emerges when the oddball deviant evokes a larger negativity than the control stimuli^99^. Next, a cluster-based permutation test was utilized to search “genuine” differential activity between the ERPs of deviant and equiprobable stimuli^124^. We conducted this analysis through the use of the Fieldtrip toolbox^125^ in MATLAB. We developed grand-averages of differential waveforms across two regions of interest (ROI) that correspond to the left (P7, PO7, O1) and right (P8, PO8, O2) posterior occipital-temporal electrodes (the electrodes were selected based on previous studies, e.g., refs. ^101,126^). For each time point (within 0–600 ms) at left or right electrodes, the clusters were formed through two or more neighboring time points whenever the *t* values (obtained by two-tailed t-test) exceeded the cluster threshold (0.025). The number of permutations was set to 10,000, and the corrected significance level was set to 0.05. That is, when the clustering level error probability of a cluster was less than 0.05, then it was considered that there were significant effects in the corresponding period (i.e., effective vMMN activities were identified). We will report the temporal range of the significant negative clusters, their mass (the sum of t values in a cluster) and the effect size of the average over the rectangular shape surrounding a cluster for each inconsistent category.

### Reporting summary

Further information on research design is available in the Nature Research Reporting Summary linked to this article.

### Supplementary information

Supplementary information

Reporting Summary

## Data Availability

The datasets generated during and/or analyzed during the current study are available upon request from the corresponding authors for non-commercial use without restriction.

## Code availability

The codes used to analyze the data are available from the corresponding authors upon request for academic purposes.
