# Supplementary material for: Seeking the neural representation of statistical properties in print during implicit processing of visual words
Source: NPJ Sci Learn. 2023 Dec 16;8:60. doi: 10.1038/s41539-023-00209-3 (PMC10724295; doi:10.1038/s41539-023-00209-3)
Supplement: Supplementary file 1 — Supplementary information [file 41539_2023_209_MOESM1_ESM.pdf]

Supplementary Table 1. Descriptive statistics of stimulus characteristics.

Stimuli	Character frequency (/Million)	Positional consistency	Phonological consistency	Semantic transparency
恨	148.87	0.7222	0.3874	High
痕	52.37	0.0556	0.3874	High
很	1545.68	0.7222	0.3874	Low
银	275.36	0.7222	0.0584	High
牲	52.75	0.5000	0.7163	High
笙	18.07	0.2500	0.7163	High
胜	246.22	0.5000	0.7163	Low
性	851.64	0.5000	0.2831	High
洋	193.13	0.7273	0.9207	High
痒	17.34	0.1818	0.9207	High
样	1964.55	0.7273	0.9207	Low
鲜	141.49	0.7273	0	High

Note: Statistic data of the characters were obtained according to a large-scale modern Chinese balanced corpus developed by Dr. Sun Maosong (Department of Computer Science and Technology, Tsinghua University). The semantic transparency of the semantic inconsistent characters were significantly lower than other characters (all *p*s<.005, Bonforroni corrected). The specific score of each character is:
恨(M=5.30; SD=1.94)、痕(M=4.46; SD=1.46)、很(M=3.18; SD=1.42)、银(M=6.27; SD=1.07); 牲(M=5.76; SD=1.23)、笙(M=5.49; SD=1.46)、胜(M=2.76; SD=0.90)、性(M=3.91; SD=1.70); 洋(M=5.91; SD=1.72)、痒(M=4.85; SD=1.46)、样(M=3.33; SD=1.67)、鲜(M=5.09; SD=1.45)

Supplementary notes

Example questions of evaluating orthographic and phonological consistency:
(1) Orthographic consistency: “To what extent do you think the position of radical "X" in the Chinese character "Y" is consistent with the position of that radical in other Chinese characters.”
(2) Phonological consistency: “To what extent do you think the pronunciation of the Chinese character "Y" composed of radical "X" is consistent with the pronunciation of other Chinese characters composed of the same radical.”
